# A Critical Functional Missense Mutation (T117M) in Sheep *MC4R* Gene Significantly Leads to Gain-of-Function

**DOI:** 10.3390/ani14152207

**Published:** 2024-07-30

**Authors:** Ziyi Zhao, Yuta Yang, Peiyao Liu, Taotao Yan, Ran Li, Chuanying Pan, Yang Li, Xianyong Lan

**Affiliations:** College of Animal Science and Technology, Northwest A&F University, Yangling, Xianyang 712100, China; zhao.ziyi@foxmail.com (Z.Z.); yydahsy@163.com (Y.Y.); liu_peiyao@126.com (P.L.); yantnt77@163.com (T.Y.); ran.li1986@hotmail.com (R.L.); chuanyingpan@126.com (C.P.)

**Keywords:** *MC4R*, function mutation, cellular mechanism, distribution, worldwide

## Abstract

**Simple Summary:**

The melanocortin 4 receptor (*MC4R*) is an important gene that can affect the growth and economic traits of sheep. This study first investigated the distribution of the *MC4R* gene’s p.T117M mutation in sheep breeds worldwide. The results showed that the mutation’s frequency was higher in European breeds compared with Chinese sheep breeds. The p.T117M mutation occurs in the first extracellular loop of *MC4R*. Mechanistically, the basal activity of the mutated receptor significantly increased. Specifically, upon treatment with α-MSH and ACTH ligands, the cAMP and MAPK/ERK signaling activation of M117 *MC4R* was enhanced. These results indicate that the T117M mutation may change the function of the gene by increasing the constitutive activity and signaling activation of cAMP and MAPK/ERK, and, thus, may regulate the growth traits of sheep. This may provide effective assistance for improving the economic benefits of the sheep industry.

**Abstract:**

The melanocortin 4 receptor (*MC4R*) gene plays a central role in regulating energy homeostasis and food intake in livestock, thereby affecting their economic worth and growth. In a previous study, the p.T117M mutation in the sheep *MC4R* gene, which leads to the transition of threonine to methionine, was found to affect the body weight at six months and the average daily gain in Hu sheep. However, there are still limited studies on the frequency of the sheep p.T117M missense mutation globally, and the underlying cellular mechanism remains elusive. Therefore, this study first used WGS to investigate the distribution of the *MC4R* gene p.T117M mutation in 652 individuals across 22 breeds worldwide. The results showed that the mutation frequency was higher in European breeds compared with Chinese sheep breeds, particularly in Poll Dorset sheep (mutation frequency > 0.5). The p.T117M mutation occurs in the first extracellular loop of *MC4R*. Mechanistically, the basal activity of the mutated receptor is significantly increased. Specifically, upon treatment with α-MSH and ACTH ligands, the cAMP and MAPK/ERK signaling activation of M117 *MC4R* is enhanced. These results indicate that the T117M mutation may change the function of the gene by increasing the constitutive activity and signaling activation of cAMP and MAPK/ERK, and, thus, may regulate the growth traits of sheep. In conclusion, this study delved into the global distribution and underlying cellular mechanisms of the T117M mutation of the *MC4R* gene, establishing a scientific foundation for breeding sheep with superior growth, thereby contributing to the advancement of the sheep industry.

## 1. Introduction

The melanocortin system encompasses a diverse array of components, comprising two antagonists, five receptors, and several agonists. The agonists, including the α-melanocyte-stimulating hormone (α-MSH), β-MSH, and γ-MSH, as well as the adrenocorticotropic hormone (ACTH), all originate from the tissue-specific post-translational processing of a pre-prohormone, pro-opiomelanocortin (POMC) [1,2]. Five melanocortin receptors (*MCR*s) mediate the varied functions of the melanocortins. They are sequentially numbered from MC1R to MC5R, mirroring the order of their cloning endeavors [2]. Notably, the melanocortin receptor subtype 4, or *MC4R*, stands as the fourth member in this repertoire of five identified receptors and is widely expressed in the central nervous system [3]. The *MC4R*, as a part of the central melanocortin system, is renowned for its mediation of orexigenic and anorexic signals in the control of appetite, food intake, body weight control, and energy homeostasis [4]. 

It is widely recognized that certain feeding conditions can trigger the expression of melanocortin, specifically, pro-opiomelanocortin (*POMC*), which is a key regulator of energy homeostasis. One of its cleavage products, alpha-melanocytotropin (α-MSH), functions as a natural agonist for the melanocortin-4 receptor (*MC4R*) [5]. The interaction between α-MSH and *MC4R* stimulates the release of thyrotropin and adrenocorticotropin-releasing hormone, resulting in a decrease in food intake among animals [6]. Furthermore, this combination inhibits the release of orexin and melanin-concentrating hormones, accelerating body metabolism, suppressing food intake, and leading to reductions in body weight and blood sugar levels [7]. Conversely, the inhibitor agouti-related peptide (AgRP) competes with α-MSH for binding to *MC4R*, inhibiting its activity and, consequently, reducing the metabolic rates of animals [6]. Moreover, *MC4R* serves as a downstream mediator in the leptin and insulin feeding regulation pathways, thereby modulating animal feeding behavior [8]. Due to its pivotal role, the *MC4R* gene is considered a significant candidate gene that is associated with mammalian growth traits. So far, several studies have reported the impact of mutations in the *MC4R* gene on crucial economic traits in animals, such as cattle [9,10], pigs [11,12,13], and sheep [14]. Research has revealed a significant correlation between the G. 1069C>G (Leu286Val) mutation in cattle and various economically important traits, including body length, chest circumference, body weight, backfat thickness, and marbling score [15,16]. In pigs, the g.1426G→A (Asp298Asn) mutation exhibits a positive correlation with several crucial traits, including carcass weight, backfat thickness, slaughter rate, meat quality score, and meat tenderness [17,18,19]. In chickens, there was a significant association between *MC4R* polymorphism and egg weight (*p* < 0.05) [20]. In sheep, a 1016G>A mutation in the 3′-UTR of *MC4R* is significantly associated with body weight, average daily gain, backfat thickness, and loin eye area [14]. Another study found that a missense mutation in the *MC4R* gene p.T117M mutation led to the transition of threonine to methionine, which was significantly associated with growth and development traits in sheep. The sheep *MC4R* gene is a G-protein-coupled receptor with 7 transmembrane structures, composed of 332 amino acid residues that are mainly involved in the regulation of body weight and energy balance [21]. Wang et al. showed that in a sheep population with the p.T117M mutation, individuals with the CT genotype exhibited significantly greater weight at 2 months of age compared to those with the TT genotype (*p* < 0.05). Furthermore, these CT individuals displayed notably longer body lengths at 4 months of age than individuals with the CC genotype (*p* < 0.05), and markedly longer body lengths than those with the TT genotype (*p* < 0.01). The 4-month-old body height of CT individuals was also significantly higher than that of CC individuals (*p* < 0.05) [22].

However, our understanding of the cellular mechanism of p.T117M missense mutation is still lacking. Therefore, this study first explored the distribution of the *MC4R* gene p.T117M in 22 sheep breeds around the world by analyzing WGS data. Subsequently, the study employed a mutation vector and a dual-luciferase reporter system to investigate the cAMP and MAPK/ERK signaling pathways regulated by *MC4R*. The objective is to unveil the molecular genetic characteristics of this crucial functional gene that impacts a sheep’s economic traits, thereby providing a theoretical foundation for the efficient breeding of economically valuable traits, the marker-assisted selection of sheep breeds, and the conservation and utilization of germplasm resources.

## 2. Materials and Methods

### 2.1. Ethics Statement

The experimental animals and procedures performed in this study were approved in accordance with the guidelines of the Faculty Animal Policy and Welfare Committee of Northwest A&F University (protocol no. NWAFU-314020038).

### 2.2. Frequency of Mutation Sites Detected by Whole-Genome Sequencing

**Figure 1:**
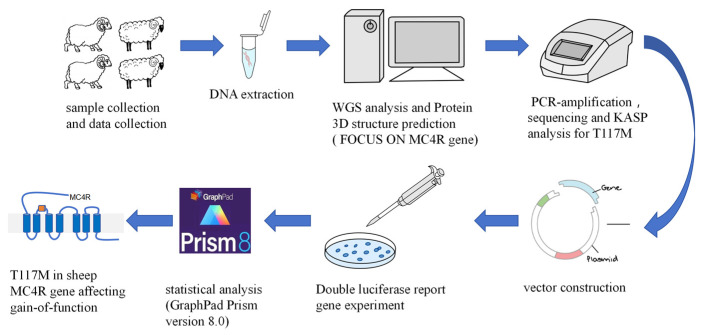
The research design.

To investigate the distribution of *MC4R* p.T117M mutations across diverse global sheep breeds, the sequencing data sets for 652 individuals representing 22 breeds from different geographic regions (China, Europe, Middle East, and Africa) were acquired through the implementation of the whole-genome sequencing (WGS) methodology. Sequencing was performed on a Sequel II instrument (PacBio) using a 30-h movie time at Annoroad Gene Technology Co., Ltd. (Beijing, China). SNP calling was performed using the “HaplotypeCaller” and “GenotypeGVCFs” modules. In addition, the detailed information regarding experiment design is illustrated in the Figure 1.

### 2.3. Protein 3D Structure Prediction

The amino acid sequence of the sheep *MC4R* gene was retrieved from the NCBI database (NP_001119842.1, GI: 187607678). Subsequently, the three-dimensional structures of both the wild-type (117T) and mutated (117M) *MC4R* proteins were predicted using the Swiss-Model tool (https://swissmodel.expasy.org/ (accessed on 20 May 2024)). For visualization of the mutant protein’s 3D structure, PyMol v3.0.3 was employed, while protscale (https://web.expasy.org/protscale/ (accessed on 20 May 2024)) was utilized to predict the physicochemical properties of the proteins in both 117M and 117T individuals.

### 2.4. Sample Collection and DNA Extraction

A total of 653 sheep from 9 breeds were selected for this study: Guiqian Semi-Fine Wool sheep (GSFW, *n* = 47, Bijie, China), Luxi Black sheep (LXBH, *n* = 91, Liaocheng, China), Lanzhou Fat Tail (LFT, *n* = 77, Lanzhou, China), Australian White sheep (AUW, *n* = 82, Tianjin, China), Yuan Sheng sheep (YS, *n* = 40, Jinchang, China), Tan sheep (*n* = 40, Yanchi, China), Mongolian sheep (*n* = 47, Mongolia, China), Weining sheep (WN, *n* = 47, China), and Hu sheep (*n* = 182, Mengjin, China). In each sheep breed, healthy sheep raised under similar feeding and management conditions were selected as test sheep. Ear tissue samples were taken from all sheep before slaughter and frozen at −80 °C to prevent degradation. Subsequently, genomic DNAs were isolated from the ear tissue samples by the high-salt extraction method, then the DNA samples were diluted to 30 ng/µL and frozen at −80 °C for further analysis [23]. The quality and purity of each DNA sample were determined by a Nanodrop 1000 (Thermo Fisher Scientific Inc., Wilmington, DE, USA).

### 2.5. Primer Design and PCR Amplification

Working according to the gene sequence of *MC4R* (NC_040274.1) listed in the Ensembl database, Primer Premier 5.0 was used to design the primer and was synthesized by Sangon Biotech (Xi’an, China). For the polymerase chain reaction (PCR), a total volume of 25 µL was used, comprising 1 µL of genomic DNA, 12.5 µL of Master Mix, 0.5 µL each of the forward and reverse primers, and 10.5 µL of ddH_2_O. The thermal cycle conditions are: 95 °C, 10 min, 95 °C, 15 s, 60 °C, 30 s, 72 °C, and 20 s, 40 cycles. The PCR products were directly confirmed by 3% agar-gel electrophoresis, and the correct amplification products were sent to Tsingke Biotechnology (Xian, China) for sequencing. The SNP sites were identified using Chromas4.1 sequence alignment software. To investigate the genetic diversity of *MC4R* p.T117M in a large population of sheep, we calculated the genotype and allele frequencies and used the Nei method to calculate the population’s genetic parameters, including homozygosity (Ho), heterozygosity (He), effective allele numbers (Ne), and polymorphism information content (PIC) [24].

### 2.6. KASP Fast Typing

To identify the missense SNP loci, KASP typing primers were specifically designed. These primers comprised a common downstream primer and two upstream primers, each with distinct 3′ terminal bases corresponding to specific alleles (Table 1). The primers were synthesized by Sangon Biotech Company (Shanghai, China). The KASP reaction comprised two key components: Primer Mix and Master Mix. The Primer Mix was a mixture of upstream and downstream primers in a ratio of 1:1:3 (FAM-labeled upstream primer: VIC-labeled upstream primer: downstream primer). The Master Mix contained two universal detection primers, each emitting a different fluorescence signal. The reaction system was formulated with 1 μL of template DNA, 6.5 μL of Master Mix, 0.5 μL of Primer Mix, and 5 μL of ddH_2_O, totaling 13 μL.

The reaction procedure was as follows: predenaturation at 95 °C for 10 min; denatured at 95 °C for 15 s; annealing at 61 °C for 45 s for 10 cycles (each cycle reduced by 0.6 °C); denatured at 95 °C for 15 s; annealing at 55 °C extended for 45 s for 28 cycles; read at 37 ° C for 1 min. After the reaction, the sample typing was judged according to the two reaction signals detected, and the different fluorescence signals obtained different genotypes.

### 2.7. Vector Construction

The mutated vector was constructed by Sangon Biotech Company. The Kozak sequence (GCCACC) was added before the N-terminal ATG of the *MC4R* gene in the sheep, and the three MYC labels sequence (GAGCAGAAGCTGATCTCAGAGGAGGACCTG) was added after the initiation codon ATG.

### 2.8. Double Luciferase Reporter Gene Experiment

In this study, the dual-luciferase reporter assay was used to detect whether these two ligands can activate downstream signals via *MC4R*. According to the PEI transfection kit (Fuseng Biotechnology Company, Shanghai, China), the luciferase reporter vector pGL4.29/pGL4.33, *MC4R* expression plasmid, and pEGFP-N1 were co-transfected into HEK-293T cells. After transfection for 24h, the cells were treated with two ligands (α-MSH, ACTH (1–24)), respectively, for 6 h, and the luciferase activity of each group was detected [25]. For each detection, two additional 48-well plates (*n* = 3) were used as technical replicates, and the data were displayed as mean ± SEM.

### 2.9. Statistical Analysis

A *t*-test or ANOVA was used to test statistical differences, and *p* < 0.05 was considered statistically significant. EC_50_ (the concentration required to induce a half-maximum effect) values were generated using GraphPad Prism version 8.0 (GraphPad Software, Inc., La Jolla, CA, USA).

## 3. Results

### 3.1. The MC4R p.T117M Mutation Is a Low-Frequency Mutation in Several Sheep Breeds

**Figure 2:**
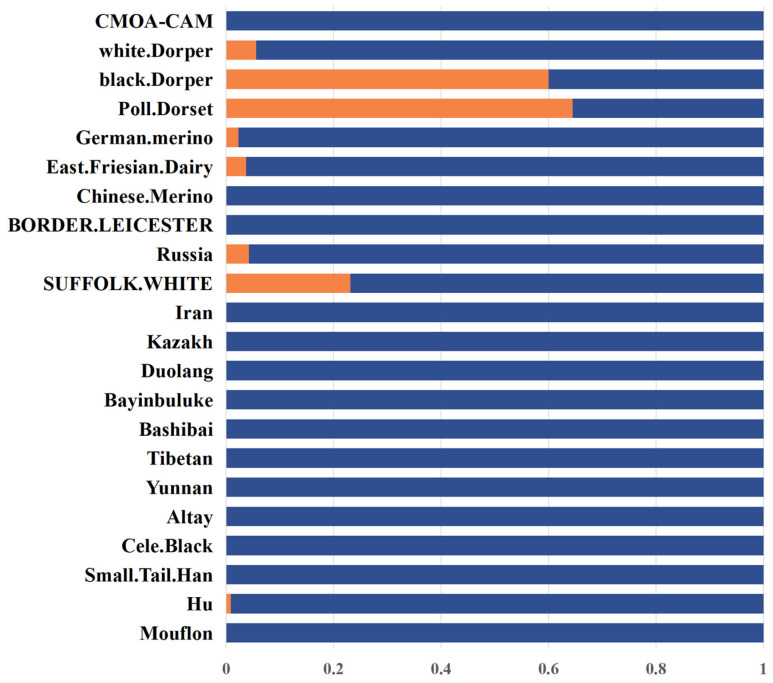
The p.T117M distribution of the *MC4R* gene in global sheep populations. The orange color represents the frequency of T117M mutation and the blue color represents the wild type frequency of this mutation.

**Figure 3:**
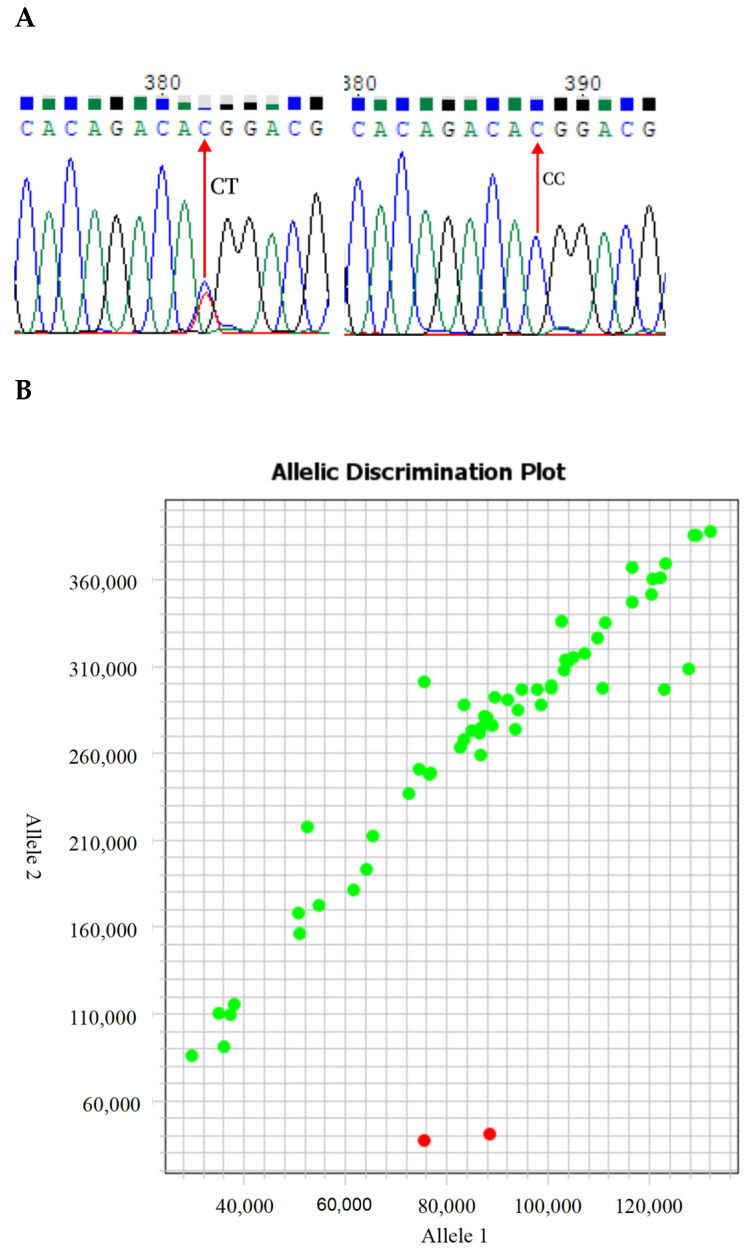
The results for the Sanger sequencing of T117M loci (**A**); cluster map of KASP genotyping at p.T117M locus in the Hu sheep population (**B**). The genotypes clustered as green circles in the upper left corner are the CC genotype, while genotypes in the lower right corner, shown as red circles, are the CT genotype.

Utilizing whole-genome sequencing (WGS) data, the allele frequencies of the p.T117M mutation across 22 diverse sheep breeds globally were analyzed, encompassing wild-type Mouflon, 10 Chinese breeds, 1 Middle-Eastern breed, 7 European breeds, and 3 African breeds. The findings are summarized in Figure 2 and Table S1 in the Supplementary Materials. Intriguingly, only 8 breeds exhibited this mutation and can be categorized into 3 distinct mutation frequency levels. Specifically, the Poll Dorset sheep from Europe and the black Dorper sheep from Africa displayed high-frequency mutations. Medium-frequency mutations were observed in the East Friesian Dairy sheep, Suffolk White sheep, Russian sheep, and German Merino sheep from Europe, as well as the white Dorper sheep from Africa. Lastly, the Hu sheep from China exhibited low-frequency mutations. To identify the influence of this mutation on the growth traits of sheep, we amplified the p.T117M site using a diagnostic PCR combined with Sanger sequencing in the five domestic sheep breeds. The PCR results showed that all individuals of AUW, WN, YS, and LXBH were detected to be in the wild-type group (CC) for p.T117M, while the CC genotype and CT genotype were detected in Hu sheep (Figure 3A). To efficiently detect this mutation in a larger population, KASP analysis was conducted on 571 individuals from 8 domestic sheep breeds. Remarkably, only two mutated individuals were identified within the Hu sheep population (Figure 3B).

The consolidated mutation detection results from both Sanger sequencing and KASP analysis (Table 2) indicate that only the Hu sheep exhibited the *MC4R* p.T117M mutation, with two distinct genotypes (CC and CT). This result is consistent with the results of the WGS analysis, indicating that the *MC4R* p.T117M mutation is a low-frequency mutation in Chinese sheep breeds. Given that the number of individuals with the mutant type in the Hu sheep breed was fewer than three, an analysis of the correlation between the p.T117M mutation and its growth traits was not performed.

### 3.2. The Schematic Structure and Hydrophilicity of Variants

**Figure 4:**
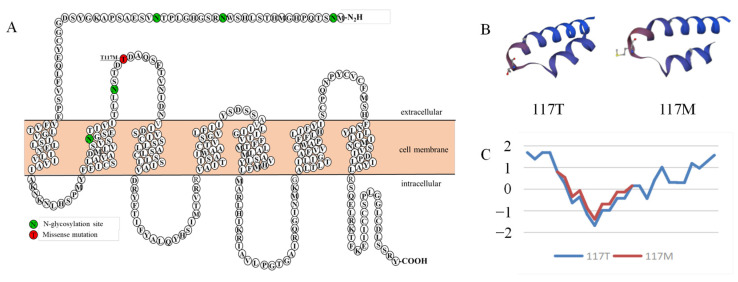
The schematic structure of *MC4R* (**A**); the influence of T117M mutation on the three-dimensional structure (**B**); changes in hydrophilicity (**C**).

To graphically illustrate the location of the mutation within *MC4R*, Figure 4A depicts the schematic structure of the receptor. Specifically, this mutation entailed a missense mutation from threonine to methionine, occurring at the first extracellular loop 117 position in the *MC4R* gene.

Utilizing PyMol software version 3.0.3, we predicted the three-dimensional protein structure of the missense mutation p.T117M. The substitution of the T117M amino acid led to a notable alteration in the local three-dimensional structure (depicted in Figure 4B), suggesting a potential functional impact on the *MC4R* gene. Furthermore, as shown in Figure 4C, this mutation resulted in a reduction in the hydrophobicity coefficient of the residue from 1.689 to 1.400, thereby increasing its hydrophilicity.

### 3.3. The T117M Mutation in MC4R Mediates Enhanced cAMP Signaling Activation

**Figure 5:**
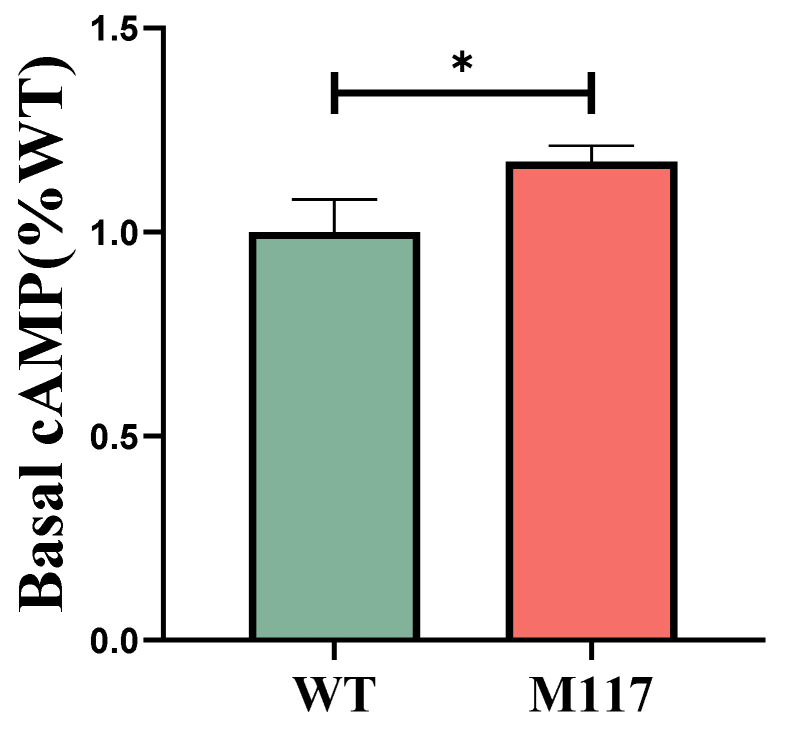
Basal cAMP (%.wt) mediated by s*MC4R* WT and mutant *MC4R*s. Comparison of basal cAMP between WT and 117M. Data are in mean ± SEM (* *p* < 0.05).

**Figure 6:**
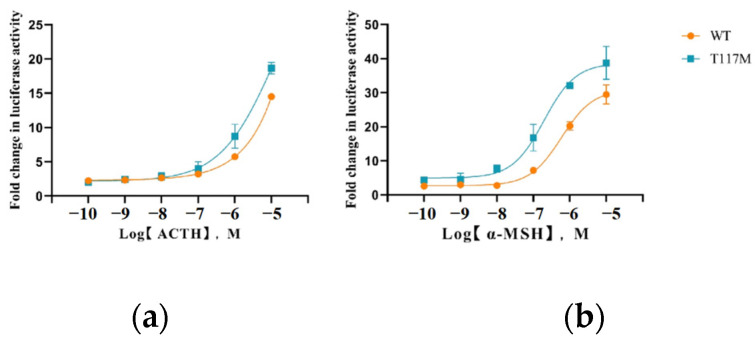
The MAPK/ERK signaling properties of WT and T117M in response to ligand stimulation. (**a**): ACTH; (**b**): α-MSH.

In this work, the HEK293T cells were transfected separately with the pGL4.29 and *MC4R*-WT/*MC4R*-T117M plasmids. Following this, the basic activity of cAMP was detected utilizing a dual-luciferase reporter system. As presented in Figure 5, a significant increase was found in the constitutive activity of the mutant (T117M) receptor, as compared to the wild-type (WT) receptor (*p* < 0.05). Furthermore, the α-MSH and ACTH ligands are used to stimulate both the WT and T117M receptors at varying concentrations, ranging from 10^−10^ M to 10^−5^ M. After 6 h, the receptor’s ability to transduce signals was assessed using the dual-luciferase reporter system. Figure 6 depicts a dose-dependent response for both receptors. Notably, under ACTH stimulation, the activity of the cAMP signaling pathway in the mutant receptor increased compared to the wild-type receptor. The semi-maximum effective concentrations (EC_50_) were calculated using GraphPad Prism 8, based on the dose-response curve. As shown in Table 3, under the stimulation of ACTH, the cAMP activity of the WT EC_50_ was about 1.5 times higher than that of T117M in activating cells (*p* < 0.05). The value was 6.124 μm for the WT receptor, whereas it was 3.933 μm for T117M, thus suggesting increased activity in the T117M cAMP signaling pathway.

### 3.4. Activation of the MAPK/ERK Pathway Mediated by MC4R WT and T117M

**Figure 7:**
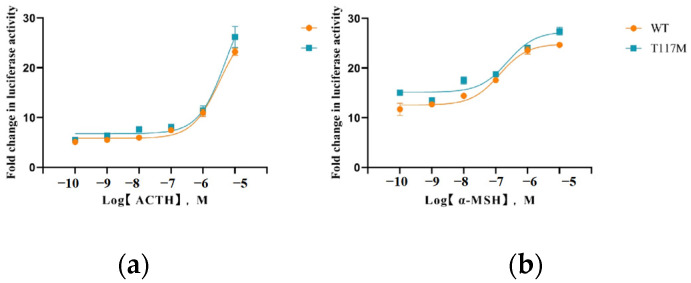
The cAMP signaling properties of WT and mutant *MC4R*s in response to ligand stimulation. (**a**): ACTH; (**b**): α-MSH.

When treated with the α-MSH and ATCH ligands, the WT and T117M receptors showed a dose-dependent stimulatory response across a concentration range of 10^−10^ M to 10^−5^ M for both ligands (Figure 7). The analysis reveals an increase in the MAPK/ERK signaling pathway activity of the mutant receptor following α-MSH stimulation. The semi-maximum effect concentrations were calculated using GraphPad Prism 8.0, based on the dose-response curve. As shown in Table 3, under the stimulation of α-MSH, the ERK1/2 activity of the WT EC_50_ was about four times higher than that of T117M in the activating cells (*p* < 0.05). The value was 0.716 μm for the WT receptor, whereas it was 0.193 μm for T117M, thus suggesting enhanced activity in the T117M MAPK/ERK signaling pathway.

## 4. Discussion

As a transmembrane neuroreceptor, *MC4R* is a downstream substance of the leptin protein that regulates feed intake and stimulates the regulatory pathway of hypothalamic neurogenic activity. *MC4R* plays an important role in mediating the function of leptin, and is mainly involved in regulating appetite, weight, energy metabolism, and other biological processes in livestock and poultry [26]. Its presence has been confirmed in pigs [27], chickens [28], sheep [29], mice [30], and other animals. Therefore, *MC4R* is considered to be an important candidate gene affecting human and animal growth and development. However, there are few reports investigating the impact of *MC4R* gene polymorphism on growth traits, as well as on the pharmacological effects of the *MC4R* gene in sheep. This study systematically studied the frequency of *MC4R* p.T117M mutations in 22 sheep populations around the world, thereby providing an important reference for using the *MC4R* mutation to improve the growth traits of various sheep breeds. Based on WGS, we statistically analyzed the allele frequency of this missense mutation in different sheep breeds around the world. According to the database, all Chinese sheep breeds except Hu sheep exhibit the C allele, while European breeds have a higher frequency of T alleles, suggesting that the mutation may have originated in Europe.

Sequencing and KASP techniques also found that the frequency of the *MC4R* p.T117M mutation was low in Chinese sheep breeds. In the tested sheep breeds, we genotyped nine sheep species from China using sequencing and KASP techniques. In this study, a total of 571 samples of 8 different sheep breeds were genotyped by KASP, and the success rate was more than 99%. The base stratification of the detection map was clear (Figure 3B) and p.T117M was detected, indicating that the KASP technique was effective and that the data thus obtained could be used for subsequent analysis. However, in this analysis of nine Chinese sheep breeds, the p.T117M mutation was only detected in the Hu sheep population, and the frequency was low. This observation is surprising and deserves further study, as a history of important overland trade should have facilitated animal exchange and gene flow between sheep breeds [31].

The primary structure of a protein determines the higher structure, which determines the function of the protein. Significant changes were found in the three-dimensional structure of the protein after the T117M mutation, suggesting that the mutation may affect the function of *MC4R*. Therefore, in order to analyze the effect of the T117M mutant on *MC4R* function in sheep, the functional differences between the T117M mutant and the wild-type receptor were analyzed for the first time in this study.

*MC4R* functions primarily via the activation of the stimulating G protein GS, ultimately resulting in an augmentation of intracellular cyclic adenosine-monophosphate (cAMP) [32]. In recent years, a growing body of research has substantiated that *MC4R* can couple to a variety of signaling pathways, such as extracellular regulated kinase (ERK) activation [33,34], and to other G proteins, such as Gi/o, Gq/11 and G12/13 [35,36]. These pathways might also be involved in the anorexigenic effect of the activated *MC4R* [37]. The receptor exhibits ligand-independent constitutive activity (basal activity) [38] and can form homodimers as well as homo-oligomers [39]. The endogenous peptide AgRP has been described as reducing this constitutive activity, i.e., acting as an inverse agonist [40] or as a competitive antagonist [41]. In addition to *MC4R* activation by endogenous ligands, *MC4R* can be activated by a variety of synthetic peptide ligands, such as (Nle4, D-Phe7)-α-MSH (NDP-α-MSH) [42].

The binding of α-MSH to *MC4R* induces stabilization of the active conformation of the receptor. The activated receptor binds to Gs, and this leads to the stimulation of adenylyl cyclase and an increased intracellular concentration of cAMP, which activates protein kinase A. The alpha-MSH-stimulated reporter gene activity was significantly reduced in cells expressing F261S *MC4R*, with a maximal response that was equal to 57% of wild-type *MC4R*. The F261S mutation also led to a significant change in the EC_50_ value compared with the wild-type receptor (*p* < 0.01) [43]. S127L has been investigated for ERK phosphorylation and was found to have increased basal activity in this pathway [44]. H158R, on the other hand, was found to exhibit reduced basal ERK phosphorylation compared to *MC4R* WT [45]. G98R was found to be a homozygous missense mutation located in its second transmembrane domain, which further leads to early-onset obesity. In vitro transient transfection assays revealed no discernable agonist ligand-binding and cAMP production in HEK293 cells expressing the mutant receptor, indicating a severe loss-of-function mutation [46]. One naturally occurring mutation, S136F, was reported to be a loss-of-function mutation with normal total and cell-surface expression, as well as normal ligand-binding properties [47]. On the first extracellular loop of *MC4R*, the T112M mutation is a loss-of-function mutation [48], suggesting that T117M may also be a loss-of-function mutation.

The signaling of ligand-receptor binding is usually the primary function of receptor proteins. α-MSH is a potent food intake inhibitor [49,50], and its ability to regulate food intake and growth in combination with *MC4R* has been demonstrated [51]. α-MSH binds to *MC4R* in the hypothalamus to reduce feeding behavior. Under the stimulation of α-MSH, our data suggest that the EC_50_ value of the MAPK/ERK signaling pathway of T117M was significantly lower than that of the wild type, indicating that the MAPK/ERK signaling pathway activities of T117M had increased. Our results show that the basal activity of cAMP had increased, suggesting that p.T117M is a gain-of-function mutation associated with growth traits. Previous studies have shown that the chest circumference of TT-genotype individuals is significantly higher than that of CT- and CC-genotype individuals and the body length of CT-genotype individuals is significantly higher than that of CC-genotype individuals [22]. However, our results show that the basal activity of cAMP increased after the mutation, suggesting that this mutation can inhibit growth.

In conclusion, this study first points out that p.T117M mutation is a gain-of-function mutation on the *MC4R* gene, providing a scientific basis for breeding sheep with good growth traits. Furthermore, the effect of this mutation on growth traits needs additional investigation.

## 5. Conclusions

In this study, a missense mutation (T117M) was detected in the coding region of the *MC4R* gene of sheep. WGS data indicated that the mutation had a higher mutation frequency in European sheep breeds and a lower mutation frequency in Chinese sheep breeds, which was further confirmed by our sequencing results and KASP typing results. The functional analysis showed that the T117M mutation significantly increased the ability to produce cAMP. Under the stimulation of the agonist, the activities of the cAMP and MAPK/ERK signaling pathway of 117M increased compared with 117T. These findings illustrate the importance of the T117M mutation in the *MC4R* gene and provide a basis for future research.

## Figures and Tables

**Table 1:** Primers sequence information.

Primers	Sequence (5′–3′)
sheep-*MC4R*-F	GGGAGGTTCAGTCAGTCCAGA
sheep-*MC4R*-R	TCTCTTAAGCTTGTGTTTGGCAC
KASP-Forward primer FAM (5′–3′)	AAGGTGACCAAGTTCATGCTTCACCCTGCTGAACAGCACAGACAC
KASP-Forward primer VIC (5′–3′)	AAGGTCGGAGTCAACGGATTTCACCCTGCTGAACAGCACAGACAT
KASP-Reverse co-primer (5′–3′)	GTCAATATTCACCGTGAAGCTCTGCGCGT

**Table 2:** Genetic diversity parameters for p.T117M in different sheep breeds.

Breeds	Genotypes (Size)	Frequency		Ho	He	Ne	PIC	χ^2^ (*p*-Value)
		Genotypes	Alleles					
Hu sheep	CC (182)	0.989	0.995 (C)	0.989	0.011	1.011	0.011	0.005 (*p* = 0.997)
	CT (2)	0.011	0.005 (T)					
	TT (0)	0						
LXBH	CC (91)	1.000	1.000 (C)	1.000	0	1.000	0	
	CT (0)	0	0 (T)					
	TT (0)	0						
LFT	CC (77)	1.000	1.000 (C)	1.000	0	1.000	0	
	CT (0)	0	0 (T)					
	TT (0)	0						
GSFW	CC (47)	1.000	1.000 (C)	1.000	0	1.000	0	
	CT (0)	0	0 (T)					
	TT (0)	0						
YS	CC (40)	1.000	1.000 (C)	1.000	0	1.000	0	
	CT (0)	0	0 (T)					
	TT (0)	0						
Tan sheep	CC (40)	1.000	1.000 (C)	1.000	0	1.000	0	
	CT (0)	0	0 (T)					
	TT (0)	0						
Mongolian sheep	CC (47)	1.000	1.000 (C)	1.000	0	1.000	0	
	CT (0)	0	0 (T)					
	TT (0)	0						
WN	CC (47)	1.000	1.000 (C)	1.000	0	1.000	0	
	CT (0)	0	0 (T)					
	TT (0)	0						
AUW	CC (82)	1.000	1.000 (C)	1.000	0	1.000	0	
	CT (0)	0	0 (T)					
	TT (0)	0						

Note: Ho, homozygosity; He, heterozygosity; Ne, effective allele numbers; PIC, polymorphism information content.

**Table 3:** The signaling properties of WT and mutant MC4R in response to ligand stimulation.

Ligand		cAMP Response	ERK1/2 Response
		EC_50_ (µM)	EC_50_ (µM)
ACTH (1–24)	WT	6.142 ± 0.835 ^a^	3.623 ± 0.303
	T117M	3.933 ± 0.255 ^b^	2.169 ± 0.919
α-MSH	WT	0.848 ± 0.443	0.716 ± 0.126 ^a^
	T117M	0.058 ± 0.009	0.193 ± 0.014 ^b^

^ab^ Different letters in the column mean statistical difference (*p* < 0.05).

## Data Availability

The data presented in this study are available in the article.

## Supplementary Materials

The following supporting information can be downloaded at: https://www.mdpi.com/article/10.3390/ani14152207/s1, Table S1: The frequencies of the p.T117M mutation in different sheep breeds.
