# Supplementary material for: A Critical Functional Missense Mutation (T117M) in Sheep MC4R Gene Significantly Leads to Gain-of-Function
Source: Animals (Basel). 2024 Jul 30;14(15):2207. doi: 10.3390/ani14152207 (PMC11311007; doi:10.3390/ani14152207)
Supplement: Supplementary file 1 [file animals-14-02207-s001.zip › animals-3118402-supplementary.pdf]

Table S1 The frequencies of the p.T117M mutation in different sheep breeds worldwide by WGS.

Species/regions	Sample size	Breeds	p.T117M
Wild	n=33	Mouflon	0.000
	n=57	Hu	0.009
	n=23	Small.Tail.Han	0.000
	n=38	Cele.Black	0.000
	n=42	Altay	0.000
China	n=21	Yunnan	0.000
	n=34	Tibetan	0.000
	n=29	Bashibai	0.000
	n=34	Bayinbuluke	0.000
	n=36	Duolang	0.000
	n=23	Kazakh	0.000
	n=13	SUFFOLK.WHITE	0.231
	n=12	Russia	0.042
	n=22	BORDER.LEICESTER	0.000
	n=39	Chinese.Merino	0.000
	n=54	East.Friesian.Dairy	0.037
	n=22	German.merino	0.023
Europe	n=31	Poll.Dorset	0.645
Africa	n=5	black.Dorper	0.600
	n=9	white.Dorper	0.056
	n=48	CMOA-CAM	0.000
Middle East	n=27	Iran	0.000
